# *Helicobacter pylori*-Mediated Protection from Allergy Is Associated with IL-10-Secreting Peripheral Blood Regulatory T Cells

**DOI:** 10.3389/fimmu.2016.00071

**Published:** 2016-03-07

**Authors:** Khiyam Hussain, Darren P. Letley, A. Borgel Greenaway, Rupert Kenefeck, Jody A. Winter, William Tomlinson, Joanne Rhead, Emily Staples, Kazuyo Kaneko, John C. Atherton, Karen Robinson

**Affiliations:** ^1^Nottingham Digestive Diseases Biomedical Research Unit, Queen’s Medical Centre, Nottingham University Hospitals, Nottingham, UK; ^2^Centre for Biomolecular Sciences, University of Nottingham, Nottingham, UK

**Keywords:** *Helicobacter pylori*, allergy, regulatory T cells, interleukin-10, IgE

## Abstract

*Helicobacter pylori* infections are usually established in early childhood and continuously stimulate immunity, including T-helper 1 (Th1), Th17, and regulatory T-cell (Treg) responses, throughout life. Although known to be the major cause of peptic ulcer disease and gastric cancer, disease occurs in a minority of those who are infected. Recently, there has been much interest in beneficial effects arising from infection with this pathogen. Published data robustly show that the infection is protective against asthma in mouse models. Epidemiological studies show that *H. pylori* is inversely associated with human allergy and asthma, but there is a paucity of mechanistic data to explain this. Since Th1 and Treg responses are reported to protect against allergic responses, we investigated if there were links between the human systemic Th1 and Treg response to *H. pylori* and allergen-specific IgE levels. The human cytokine and T-cell responses were examined using peripheral blood mononuclear cells (PBMCs) from 49 infected and 58 uninfected adult patients. Concentrations of total and allergen-specific plasma IgE were determined by ELISA and ImmunoCAP assays. These responses were analyzed according to major virulence factor genotypes of the patients’ colonizing *H. pylori* strains. An *in vitro* assay was employed, using PBMCs from infected and uninfected donors, to determine the role of Treg cytokines in the suppression of IgE. Significantly higher frequencies of IL-10-secreting CD4^+^CD25^hi^ Tregs, but not *H. pylori*-specific Th1 cells, were present in the peripheral blood of infected patients. Total and allergen-specific IgE concentrations were lower when there was a strong Treg response, and blocking IL-10 *in vitro* dramatically restored IgE responses. IgE concentrations were also significantly lower when patients were infected with CagA^+^ strains or those expressing the more active i1 form of VacA. The systemic IL-10^+^ Treg response is therefore likely to play a role in *H. pylori*-mediated protection against allergy in humans.

## Introduction

*Helicobacter pylori* infection usually becomes established during early childhood (1), when the immune system is developing, and it persists life-long in the absence of effective treatment (2). Peptic ulceration and gastric malignancy may result; however, the infection is asymptomatic in the vast majority of cases. In recent years, there has been considerable interest in the possible beneficial effects of *H. pylori* infection (3–6). Protective associations between the infection and risk of atopy, asthma, and autoimmunity have been reported in epidemiological studies by us and several other groups (7–21). Not all studies have been able to demonstrate such trends, however (22–24). A meta-analysis of 700 cases and 785 controls could not prove a link between *H. pylori* infection and asthma risk (25), and some researchers remain skeptical (26). The most consistently observed protective associations with asthma and atopy, however, are in children (8, 16, 18, 20, 24, 27).

The incidence of atopic disease in developed countries has increased markedly over the past 50 years (28, 29). Although genetic predisposition is very important, genetic changes cannot explain this recent dramatic trend. The worldwide prevalence of *H. pylori* is declining, and fewer children are now infected (6, 30, 31). In developing countries, such as India and Mexico, the infection remains present in over 80% of the population, but in many developed countries, the prevalence of *H. pylori* is now less than 20% and it is expected to decline further (32, 33). A role for *H. pylori* in the “hygiene hypothesis” has been suggested, where childhood exposure to certain infections is needed for development of a healthy immune system (34, 35). Modernization has diminished exposure to many of the immunoregulatory stimuli that humans have co-evolved with, including intestinal parasites, ectoparasites, environmental bacteria, gut commensal organisms, and also *H. pylori* (10, 35–38). It is thought that during the past 60,000 years, human physiology has developed with the continual presence of the bacterium in the stomach (6, 39). There is growing evidence that adverse consequences may arise from a lack of exposure to *H. pylori* (5).

Allergies occur more commonly when certain immunological exposures are absent. Mechanisms include the stimulation of ­regulatory T cell (Treg) and T-helper 1 (Th1) responses to counterbalance and suppress Th2 activity in atopy (37, 38, 40–42). Although the epidemiological evidence for protective associations of *H. pylori* infection with atopy is compelling, it could be argued that the infection is simply a marker for other exposures with similar risk factors. The first indication of a causal relationship came from finding stronger links between childhood asthma and infection with more pathogenic CagA^+^ *H. pylori* strains (9). Direct proof of *H. pylori*-mediated protection in humans is still lacking, however. Arnold et al. were the first to demonstrate in a mouse model that *H. pylori* infection is protective against allergic asthma (43). In agreement with the human epidemiological data, these effects were stronger when the mice were infected as neonates. The mechanism was demonstrated to involve dendritic cell-mediated induction of immunosuppressive regulatory T-cells (Tregs), and the *H. pylori* factors important in driving this include vacuolating cytotoxin A (VacA), gamma glutamyl transpeptidase (GGT), and urease (44–47).

There is a wealth of published data demonstrating that *H. pylori* infection induces high-level Treg, Th1, and Th17 responses in both humans and mouse models (48–53). Interferon-gamma (IFNγ)-secreting Th1 cells are associated with increased gastric inflammation and disease, whereas Tregs inhibiting inflammation are associated with reduced incidence of disease and probably contribute to the life-long persistence of *H. pylori* infections (32, 48). They are known to be important for suppressing autoimmunity, allergy, and inflammation (54, 55). There are several different types of Treg cells, commonly characterized as CD4^+^, FOXP3^+^, CD127^low^, and expressing high levels of CD25 (56). Tregs may act *via* a number of mechanisms, including contact-mediated suppression, or secretion of suppressive cytokines, such as interleukin-10 (IL-10), IL-35, or transforming growth factor beta (TGFβ) (57–59). IL-10 is known to be protective against allergy, acting *via* inhibition of antigen presentation, down-regulation of effector T-cell cytokine expression, and inhibition of mast cell degranulation (42, 60–62). TGFβ is also reported to play a role in protection against some animal models of allergy (63, 64). Because of the ontogeny of the immune system, *H. pylori*-infected children tend to have stronger Treg responses than adults (65–67). This aligns with stronger protection in the neonatally infected mouse allergy model (43) and also raises the hypothesis that the elevated frequencies of Tregs present in the circulation of *H. pylori*-infected humans (68) play an important role in protection against allergy.

While a great deal of convincing animal model data exist, it has frequently been difficult to prove protective effects of infections, including parasites, on clinical atopy and asthma (69). For this reason, we decided to look for more subtle effects, studying the IgE responses of non-atopic, non-asthmatic patients with carefully characterized *H. pylori* infection status. As reported previously (68), we found higher frequencies of IL-10-secreting Tregs in the peripheral blood of infected patients. Very low plasma IgE concentrations were present in those with the highest Treg responses, but there were no associations with the Th1 response. This indicated that higher levels of *H. pylori*-induced Tregs are associated with reduced markers of allergy. *In vitro* experiments also confirmed that blocking IL-10 (but not IFNγ or TGFβ) resulted in significantly increased IgE secretion by peripheral blood mononuclear cells (PBMCs) from infected, but not uninfected, donors. These data indicate a role for *H. pylori*-induced IL-10-secreting Tregs in protection against human allergy.

## Materials and Methods

All reagents were obtained from Sigma-Aldrich Ltd. (Poole, UK) unless otherwise stated.

### Ethics Statement

Clinical samples were donated with informed written consent and approval from Nottingham Research Ethics Committee 2 (reference 08/H0408/195).

### Volunteers and Clinical Materials

Samples were donated by 107 patients (aged 19–83 years) undergoing a routine upper gastro-intestinal endoscopy at the Queen’s Medical Centre, Nottingham, for a variety of indications (most commonly dyspepsia), but were otherwise healthy. Out of 107 patients, 49 donors were *H. pylori*-positive (14 duodenal ulcer, 9 gastric ulcer, and 26 no ulceration) and 58 were negative (none had peptic ulcers). Male-to-female ratios of these groups were 0.93 and 0.70, respectively; mean ages were 59.7 and 54.6 years, respectively. None of the patients were regularly taking non-steroidal anti-inflammatory drugs or had taken antibiotics or proton pump inhibitor drugs in the preceding 2 weeks. None had clinically diagnosed asthma or allergies.

A series of biopsies were collected from the gastric antrum for determination of *H. pylori* infection status by urease detection, isolation and culture of the organism, and histopathology (48). DNA was purified from *H. pylori* isolates and PCR-genotyping carried out to ascertain *cagA* and *vacA* genotype status (70, 71). The 20-ml peripheral blood samples were collected into EDTA vacutainers. Samples of plasma were separated and stored at −80°C. PBMCs were purified by density gradient centrifugation using Histopaque1077.

### Quantification of Treg and Th1-Associated Cytokine Responses of Human PBMCs

Peripheral blood mononuclear cells at 1 × 10^6^/ml in AIM V medium (Invitrogen) were placed 0.2 ml/well into 96-well plates in duplicate. A *H. pylori* lysate antigen prepared from six clinical isolates (48) was added at 25 μg/ml. Negative controls received no antigen, whereas positive controls received 5-μg/ml concanavalin A (conA) before incubation for 48 h at 37°C in 5% CO_2_. Commercial ELISA kits (eBioscience, Hatfield, UK) were used to quantify IL-10 and TGFβ1 in culture supernatants, as per the manufacturer’s instructions.

### Quantification of Treg and Th1 cells in Human PBMCs

As described previously (48, 68, 72), 1 × 10^6^ PBMCs were placed into culture tubes in RPMI1640 with 10% fetal calf serum (FCS), 100 U/ml penicillin, and 100 μg/ml streptomycin. *H. pylori* lysate antigen was added at 25 μg/ml, negative controls received no antigen, and positive controls received 20 ng/ml phorbol myristate acetate (PMA) and 1 μmol/l ionomycin. Brefeldin A (BFA) was added to all tubes at 10 μg/ml before 16 h incubation at 37°C in 5% CO_2_.

Cells were stained with anti-CD4-phycoerythrin-Texas Red (ECD; Beckman Coulter UK Ltd., High Wycombe, UK) and anti-CD25-PE-cyanin 5 (PC5; Biolegend, Cambridge BioScience Ltd., Cambridge, UK), with either anti-CD127-phycoerythrin (PE; eBioscience, San Diego, CA, USA), anti-CTLA-4-PE (Beckman Coulter), or anti-GITR-PE (Biolegend) antibodies in individual tubes. Fixed and permeabilized cells were stained with anti-IL-10-PE (AbD Serotec, Oxford, UK) and anti-FOXP3-Alexa Fluor^®^488 antibody conjugate (Biolegend). The gating of CD25^hi^ was set based on the positions of the FOXP3^+^ and CD127^lo^ populations in CD4^+^ events. Alternatively, cells were stained with anti-CD4-ECD and fixed in 0.5% formaldehyde before permeabilization and staining with anti-CD69-PC5 and anti-IFNγ-FITC (Beckman Coulter).

Data on 200,000 events were acquired using a Beckman Coulter EPICS Altra flow cytometer and analyzed using Weasel version 3 (73), with reference to appropriate isotype controls.

### *FOXP3* and *IL10* RT-qPCR

The 5 × 10^6^ PBMCs were purified from fresh blood samples using Histopaque 1077, and total RNA was extracted using an RNeasy minikit (QIAGEN, Crawley, UK), as per the manufacturer’s instructions. cDNA was synthesized using SuperScript II reverse transcriptase with an oligo(dT) primer (Invitrogen). Real-time PCR was performed on a Rotor-Gene 3000 (Corbett Research, Cambridge, UK) using primers described in Table 1, with a DyNAmo HS SYBR green qPCR kit (New England Biolabs, Ipswich, UK) as previously described (48). Amplification was over 45 cycles of 15 s at 95°C, 30 s at 62°C, and 30 s at 72°C. No template controls were included in each run, and a melting curve analysis was performed. First-stage RT-qPCR samples, produced without reverse transcriptase, were assayed in parallel. Results were analyzed using the Pfaffl method (74). *FOXP3* and *IL10* expression levels were normalized against *GAPDH*, comparing to a pooled reference sample from five *Hp*-negative donors to obtain a fold difference. A commercial pooled human cDNA standard (BD Biosciences) was included as a positive control in all assays.

**Table 1:** **Primer sequences for real-time PCR**.

	Primer	
Gene	Forward	Reverse
*GAPDH*	CCACATCGCTCAGACACCAT	GGCAACAATATCCACTTTACCAGAGT
*FOXP3*	CAGCACATTCCCAGAGTTCCT	GCGTGTGAACCAGTGGTAGAT
*IL10*	GCTGGAGGACTTTAAGGGTTACCT	CTTGATGTCTGGGTCTTGGCT

*Sequences are shown in the 5′–3′ orientation*.

### Human IgE Assays

Plasma IgE concentrations were determined using a Human IgE ELISA Quantification Kit (Bethyl, USA) as per the manufacturer’s instructions. Optical densities were measured at 450 nm with a reference wavelength of 595 nm. Concentrations of IgE were determined from a standard curve on each plate. Allergen-specific IgE assays [timothy grass pollen, birch tree pollen, and *Dermatophagoides pteronyssinus* (house dust mite)] were carried out by the Department of Immunology, Queen’s Medical Centre, Nottingham, using the ImmunoCAP system (Phadia AB, Uppsala, Sweden).

### *In Vitro* PBMC Culture and Manipulation of the Cytokine Response

A method to induce IgE synthesis in PBMC cultures was adapted from previously published studies (75–77). PBMCs from 5 *H. pylori*-positive and 10 *H. pylori*-negative healthy non-allergic donors were resuspended in RPMI 1640 medium (supplemented with 10% FCS, 100 U/ml penicillin, 100 μg/ml streptomycin, 50 μg/ml transferrin, and 4 μg/ml insulin). The 200-μl aliquots containing 3 × 10^5^ cells were added to the wells of a sterile 96-well polystyrene round-bottomed plate (Thermo Scientific). In order to induce Th2-skewed conditions, rIL-4 and sCD40L were added to the cells at final concentrations of 50 μg/ml and 100 ng/ml, respectively (Peprotech). The PBMCs were cultured for 12 days at 37°C and 5% CO_2_. Supernatants were then removed and stored at −80°C.

In order to observe the effects of IL-10 and TGFβ on IgE synthesis, on day 0 of the culture, 20-μl recombinant human IL-10 (rIL-10) (AbD Serotec) or rTGFβ (AbD Serotec) were added at various concentrations to appropriate wells in quadruplicate (76). The equivalent volume of medium was added to control wells. Cytokines were blocked by adding anti-IL-10 (rat IgG1 clone JES3-9D7, eBioscience) and anti-TGFβ (mouse IgG1 clone 1D11, Abcam) mAbs at varying concentrations. The respective isotype control antibodies were added at the equivalent concentrations. The 20-μg/ml JES3-9D7 has previously been shown to efficiently neutralize endogenously produced IL-10 in a similar culture system (77). The neutralization dose (ND50) of 1D11 antibody is typically 0.25–1.25 μg/ml in the presence of 1 ng/ml recombinant human TGFβ1 (78). The PBMCs were then cultured for 12 days at 37°C with 5% CO_2_ before collection and assay of the supernatants for IgE.

### Statistical Analysis

Statistical analyses were carried out using Prism 6.00 (GraphPad Software, CA, USA). Since the data were not normally distributed, non-parametric analysis methods were used. Statistical tests of paired data were carried out using the Wilcoxon signed rank test. For unpaired data, the Mann–Whitney *U*-test was used. A significant difference was taken at *p* ≤ 0.05.

Box and whisker plots depict the medians and interquartile ranges in the boxes, with whiskers extending to the maximum and minimum points in the data.

## Results

### Peripheral Blood Th1 and Treg Responses in Infected and Uninfected Patients

**Figure 1:**
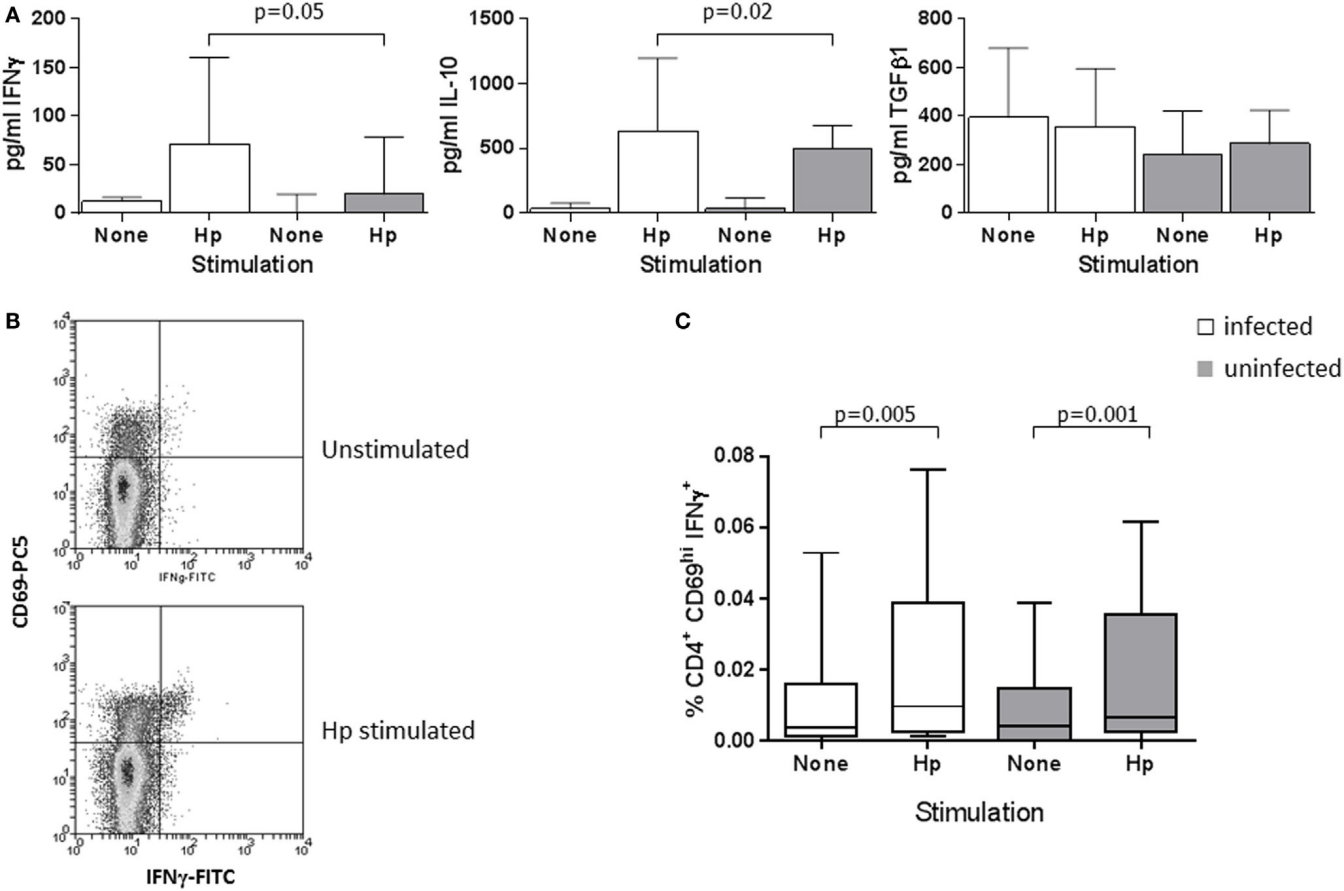
**Peripheral blood Th1 and Treg cytokine responses in *H. pylori*-infected and uninfected patients**. **(A)** PBMCs from 13 infected patients (white bars) and 11 uninfected patients (gray bars) were cultured for 48 h in the presence of 25 μg/ml *H. pylori (Hp)* lysate, with medium alone as a negative control or with 20 μg/ml conA as a positive control stimulus. IFNγ, IL-10, and TGFβ1 concentrations were determined using commercial ELISA kits. Bars depict the median and error bars show the 95% confidence interval. Following conA stimulation, median concentrations from the PBMCs of infected and uninfected patients were, respectively, 1885 and 2035 pg/ml IFNγ, 375 and 393 pg/ml IL-10, and 352 and 306 pg/ml TGFβ1. Th1 cells were quantified in PBMCs from 34 infected and 24 uninfected patients using flow cytometry. **(B)** Example flow cytometry dot plots of gated CD4^+^ cells from an infected donor, stained for CD69 and IFNγ after culture of PBMCs in medium alone, or with *Hp* lysate antigen. Quadrant indicates the position of CD69^hi^ IFNγ^+^ events, with the lower left quadrant containing both CD69-negative and -positive events. **(C)** The percentage of CD4^+^CD69^hi^ IFNγ^+^ events among PBMC lymphocytes is shown following culture in medium alone (unstimulated) or with *Hp* lysate antigen. When stimulated with PMA and ionomycin as a positive control stimulus, the median percentages of CD4^+^CD69^hi^IFNγ^+^ events in PBMCs from infected and uninfected donors were 1.13 and 1.17%, respectively. The percentages of CD4^+^CD69^hi^IFNγ^+^ events were not significantly different between *Hp*-stimulated samples from infected and uninfected donors.

Previous work showed a high-level Treg and Th1 response in the *H. pylori*-infected human gastric mucosa and significantly increased numbers of CD4^+^CD25^hi^ Tregs in peripheral blood (48, 68). We further investigated the circulating Treg population and determined if higher numbers of Th1 cells were also present. We began by quantifying the cytokines IFNγ, IL-10, and TGFβ1 in PBMC culture supernatants from infected and uninfected patients, and comparing the frequencies of Treg and Th1 cells by flow cytometry. PBMCs from all patients secreted IFNγ when stimulated with *H. pylori* lysate antigen (*Hp*) (Figure 1A) or the positive control mitogen conA; however, the concentrations were higher (median difference: 3.6-fold) among the *Hp*-stimulated supernatants from infected compared to uninfected individuals (*p* = 0.05). This is indicative of a systemic Th1 response to *Hp* infection. Similarly, there were slightly higher concentrations of IL-10 (median: 1.3-fold, *p* = 0.02), but there were no differences in TGFβ1 levels.

Using flow cytometry to quantify *Hp*-specific Th1 cells, we immunostained for the early activation marker CD69 (72) and analyzed cells that were activated by *Hp* lysate antigen (Figure 1B). Higher frequencies of CD69^hi^CD4^+^ events were obtained from the *Hp*-stimulated PBMCs of infected compared to uninfected donors (median: *Hp*^+^ 10.1%, *Hp*^−^ 4.9%; *p* = 0.001), demonstrating a higher frequency of *Hp*-specific systemic T-cells during infection (data not shown). When cells from both infected and uninfected donors were stimulated with *Hp*, there was a significantly increased frequency of CD69^hi^CD4^+^IFNγ^+^ events compared to unstimulated PBMCs (paired analyses: infected *p* = 0.005; uninfected *p* = 0.001; Figure 1C), and similar statistically significant trends were found following PMA/ionomycin stimulation. No significant differences in the responses of cells from infected and uninfected donors were found; therefore, we could not confirm an increased frequency of Th1 cells in the peripheral blood of the infected patients. There were also no differences according to the *cagA* virulence genotype of the colonizing bacterial strains (not shown).

**Figure 2:**
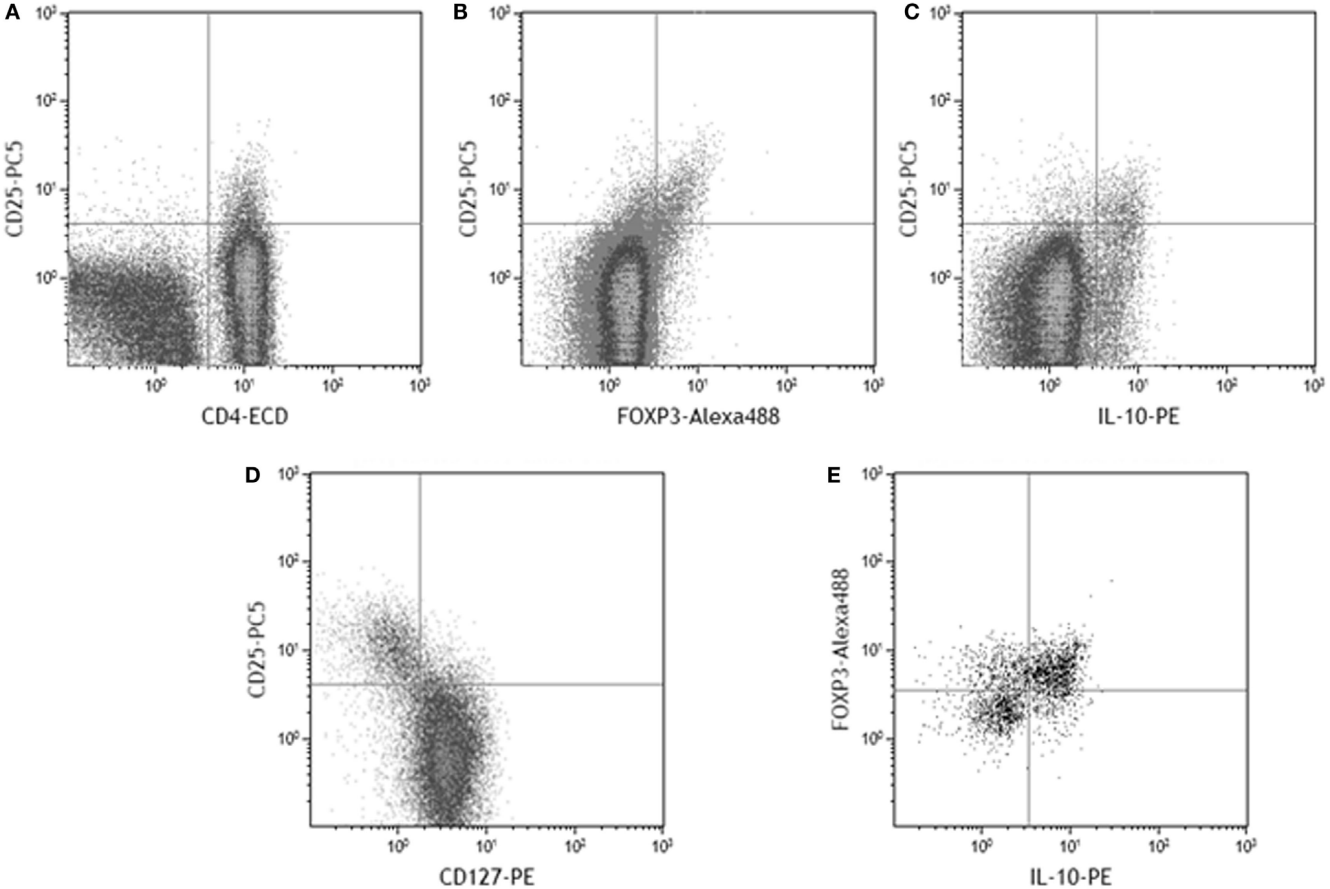
**Example flow cytometry dot plots to display the gating and designation of CD25^hi^ cells among PBMC lymphocytes**. CD25^hi^ events were gated on the basis that expression is of higher intensity in CD4^+^ events **(A)**. Among gated CD4^+^ events, the CD25^hi^ population contains FOXP3^+^ events **(B)**, IL-10^+^ events **(C)**, and corresponds to the CD127^lo^ population **(D)**. Among gated CD4^+^CD25^hi^ events, the majority of IL-10^+^ events were also FOXP3^+^ **(E)**.

**Figure 3:**
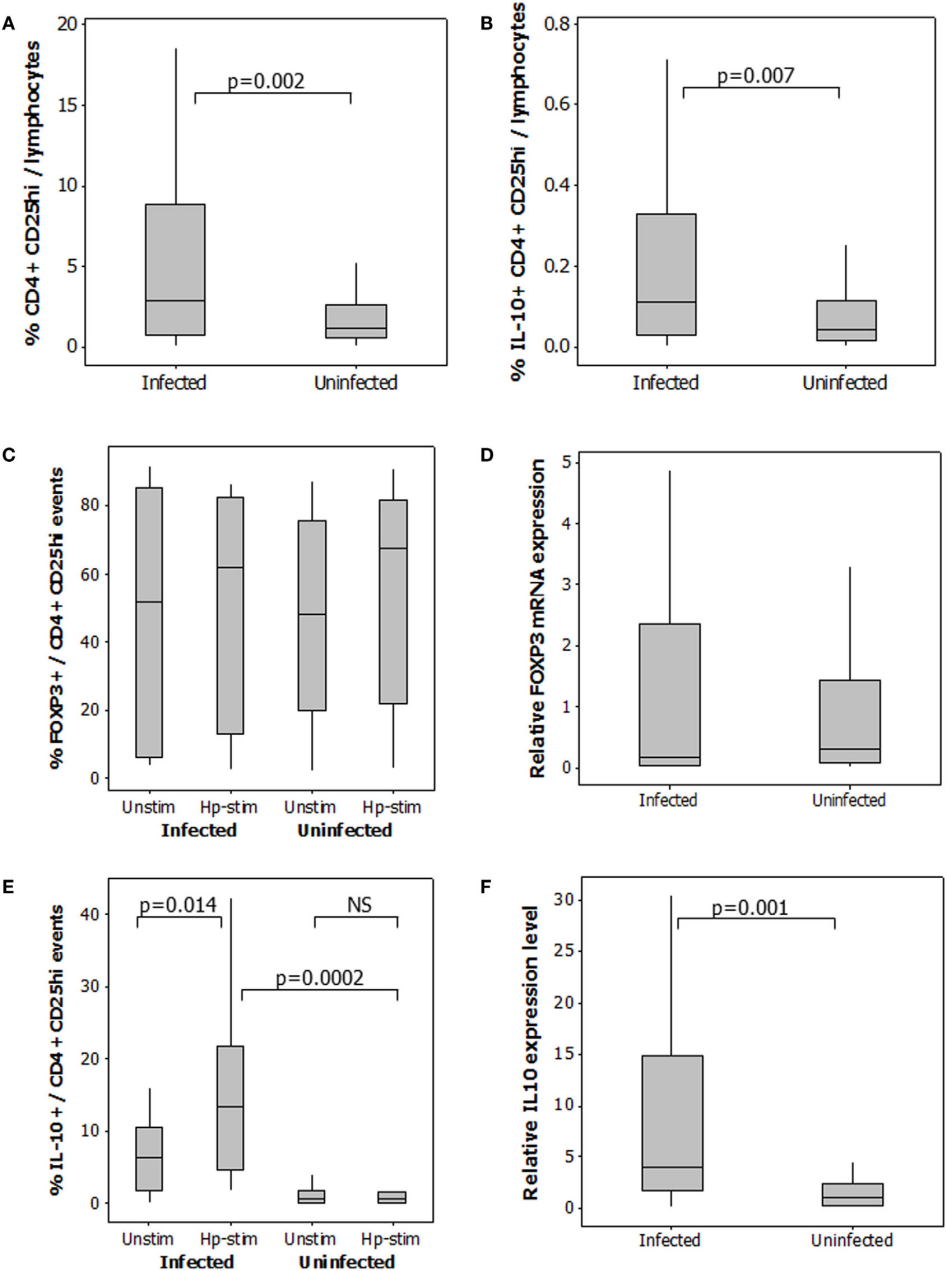
**Regulatory T-cells in the peripheral blood of 49 *H. pylori*-infected and 58 uninfected patients**. The percentage of CD4^+^CD25hi events among gated lymphocytes **(A)**. For determination of IL-10^+^CD4^+^CD25^hi^ Tregs **(B)**, PBMCs were cultured in the presence and absence of *Hp* lysate antigen. Median frequencies of IL-10^+^CD4^+^CD25^hi^ events among unstimulated cells were 0.04% (uninfected donors) and 0.04% (infected donors). The proportion of CD4^+^CD25^hi^ cells expressing FOXP3 **(C)** and IL-10 **(E)** was assessed in PBMCs cultured with and without *Hp* lysate antigen (Unstim and Hp-stim, respectively). RT-PCR was performed on freshly isolated PBMCs from 48 infected and 26 uninfected patients to quantify *FOXP3* **(D)** and *IL10* **(F)** mRNA expression levels. Data were normalized relative to *GAPDH* expression. NS = no significant difference.

To quantify the Treg response, CD25^hi^ staining was taken as a higher intensity of fluorescence compared to that observed for CD4 negative cells (Figure 2A), included the FOXP3^+^ and IL-10^+^ populations (Figures 2B,C), and corresponded with the CD25 staining intensity in the CD127^lo^ population (Figure 2D). As expected, not all CD4^+^CD25^hi^ events expressed FOXP3. 48–81% of gated CD4^+^CD25^hi^IL-10^+^ events were FOXP3^+^ (Figure 2E). A 2.5-fold higher median frequency of CD4^+^CD25^hi^ cells (*p* = 0.002) was present in PBMCs from infected patients (Figure 3A). There was also a 2.5-fold increased level of IL-10^+^ CD4^+^CD25^hi^cells (*p* = 0.007; Figure 3B). There was a modest 1.7-fold increase in the frequencies of CD4^+^CD25^hi^CTLA-4^+^ cells (*p* = 0.007, data not shown), but there was no difference in the frequencies of CD4^+^CD25^hi^GITR^+^ cells (not shown). The proportion of FOXP3^+^ events among CD4^+^CD25^hi^ cells was not different between infected and uninfected donors, or with/without antigen stimulation (Figure 3C), and *FOXP3* mRNA levels were no different when assaying PBMCs by RT-qPCR (Figure 3D). The variation in FOXP3^+^ cell frequencies was extremely wide within both the infected and the uninfected groups, which hampered our ability to detect statistically significant differences. The frequencies of IL-10^+^ and FOXP3^+^ CD4^+^CD25^hi^ cells and the corresponding *IL10* and *FOXP3* transcript levels did not vary significantly according to the age or gender of the patient groups; however, those with gastric or duodenal ulcers had significantly lower frequencies of CD4^+^CD25^hi^ cells than who only had gastritis (medians: 1.80 and 3.89%, respectively; *p* = 0.047). Similar findings were obtained when analyzing CD4^+^CD25^hi^IL-10^+^ data (medians: 0.10 and 0.17%, respectively; *p* = 0.041). In contrast, the proportion of FOXP3^+^ events among CD4^+^CD25^hi^ cells was not significantly different with respect to peptic ulcer disease status among the infected group. Stimulating PBMCs from infected patients with *Hp* antigen resulted in a significant increase in the proportion of Tregs secreting IL-10 (*p* = 0.01), but there was no significant effect on cells from uninfected patients (Figure 3E). This indicates that *H. pylori* infection induces a specific IL-10-secreting peripheral blood Treg response. *IL10* mRNA levels in freshly isolated PBMCs from infected patients were 3.8-fold higher than the uninfected group (*p* = 0.001; Figure 3F), and there was a 4.8-fold higher mRNA level in the samples from infected patients without peptic ulcer disease compared to those who had ulcers (*p* = 0.017). These data confirm the presence of increased numbers of IL-10^+^ cells in the circulation of *H. pylori*-infected patients, particularly when peptic ulceration is absent. Since there was an elevated systemic IL-10-secreting Treg response in infected patients, we hypothesized that as in the mouse, this could be an important mechanism behind the protective associations with allergy.

### Association between the Human Regulatory T-Cell and IgE Responses

**Figure 4:**
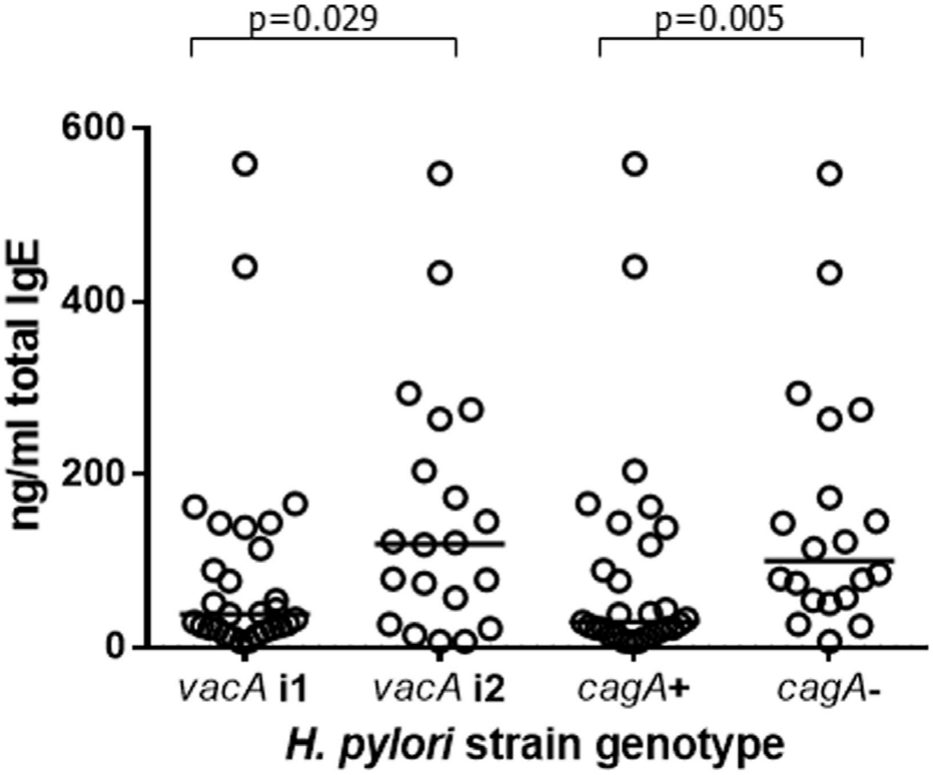
**Plasma IgE concentrations from 49 patients infected with *H. pylori* strains of differing virulence genotypes**. Of the 29 *cagA*^+^ strains, 24 were of the i1 *vacA* genotype and 5 were *vacA* i2. Of the 20 *cagA*^−^ strains, 15 were *vacA* i2 and 5 were of the *vacA* i1 genotype. Points represent the results from individuals and horizontal lines depict the median.

Total and allergen-specific IgE concentrations in the plasma of 49 infected and 46 uninfected patients were not significantly different (Table 2); however, among the infected group, there were differences in total IgE according to the virulence genotype of the colonizing *H. pylori* strains. This was important to evaluate, since presence of a strain expressing CagA has previously been reported to be associated with greater protection against asthma (9). VacA is known to be protective against allergy in mice (45). Since *cagA*^+^ strains usually also express the most active i1 form of *vacA* (71), we investigated how IgE levels related to these virulence factors. Apart from in five cases, the 29 *cagA*^+^ strains were also of the more active *vacA* i1 type. All but 5 of the 20 less pathogenic *cagA*^−^ strains were typed as having the less active i2 form of *vacA*. We were therefore unable to pick apart the roles of these factors individually. The patients infected with the more virulent *cagA*^+^ or *vacA* i1 strains had threefold lower concentrations of IgE compared to those with *cagA*^−^ or *vacA* i2 strains (Figure 4; *p* = 0.005 and *p* = 0.029, respectively). Surprisingly, however, there were no significant differences in Treg frequencies or IL-10 transcripts between those with *cagA*^+^ versus *cagA*^–^ strains, and the same result was found for *vacA* genotypes (data not shown).

**Table 2:** **Total and allergen-specific IgE levels in the plasma of 49 infected and 46 uninfected patients**.

IgE measurement	Median concentration and interquartile range	
	*H. pylori*-positive	*H. pylori*-negative
Total (ng/ml)	58.7 (22.1–140.1)	31.4 (9.61–133.3)
House dust specific (kUA/l)	0.05 (0.03–0.13)	0.04 (0.02–0.21)
Grass pollen specific (kUA/l)	0.00 (0.00–0.03)	0.00 (0.00–0.03)
Birch pollen specific (kUA/l)	0.01 (0.00–0.02)	0.00 (0.00–0.01)

**Figure 5:**
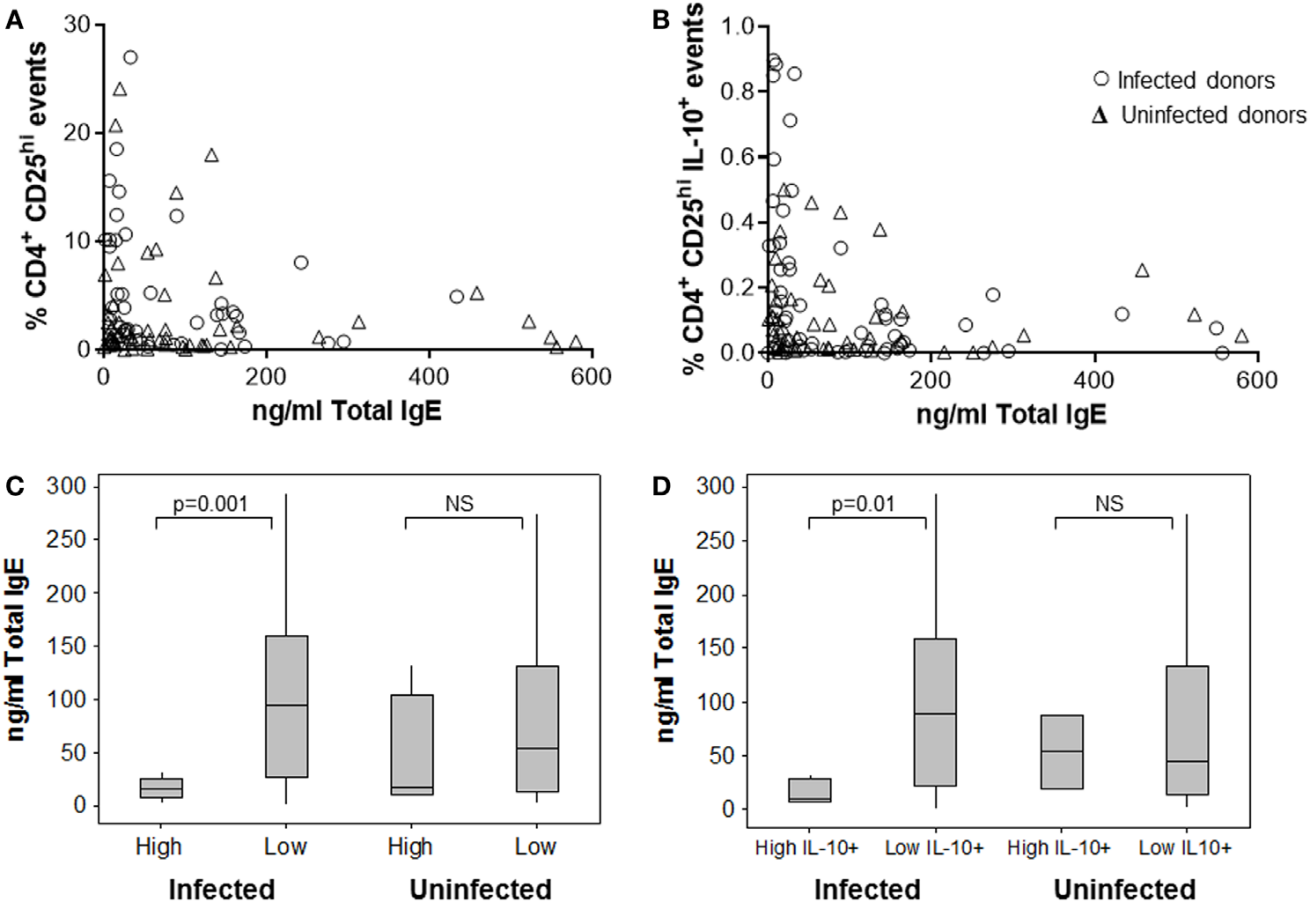
**Association between human peripheral blood regulatory T-cells and the IgE response**. The frequency of CD4^+^CD25^hi^ **(A)** and IL-10^+^ CD4^+^CD25^hi^ Tregs **(B)** in PBMCs from 49 infected and 46 uninfected donors was assessed by flow cytometry after 15 h culture with *Hp* lysate antigen. This was compared to the total plasma IgE concentration for each patient. The data were divided according to whether there were high (>10%) or low frequencies of CD4^+^CD25^hi^ lymphocytes **(C)** and high (>0.4%) or low frequencies of IL-10^+^CD4^+^CD25^hi^ events **(D)** to further examine associations with total IgE concentrations. NS = no significant difference.

When comparing the magnitude of the Treg response with IgE levels, we observed some interesting trends. Patients with the highest frequencies of CD4^+^CD25^hi^ PBMCs had the lowest total IgE concentrations. The effect was stronger among the samples from *H. pylori*-positive patients (Figures 5A,C), where having a high-level Treg response was more common. The data were stratified according to whether or not CD4^+^CD25^hi^ cell frequencies were above or below 10%, which equated to the mean + 1 SD of all samples tested and was well above the normal range for frequencies of these cells in peripheral blood as previously reported in other studies (79, 80). Among the samples from infected patients with high CD4^+^CD25^hi^ cell frequencies, there was a fivefold lower median IgE concentration, compared to those with frequencies below 10% (*p* = 0.001) (Figure 5C). Among the samples from infected patients with high IL-10^+^ CD4^+^CD25^hi^ cell frequencies (>0.4%, mean + 1 SD), similarly there was a fivefold lower median IgE concentration, compared to those with low IL-10^+^ Treg frequencies (*p* = 0.01) (Figure 5B,D). There was no significant difference in the total plasma IgE concentrations of uninfected patients with high and low Treg frequencies (Figures 5C,D).

A significantly reduced grass (*p* = 0.004) and birch (*p* = 0.002) pollen-specific IgE concentration was present among infected patients with high frequencies of peripheral blood Treg cells (Table 3), but there were no significant differences among the uninfected group, or with regard to house dust mite-specific IgE. Similar trends were observed when comparing IgE concentrations with frequencies of IL-10^+^CD4^+^CD25^hi^ PBMCs (data not shown). There were no significant trends when PBMC Th1 responses were compared with IgE levels (data not shown). Together, these data indicate that *H. pylori* infection is associated with unusually high circulating Treg levels, which could perhaps directly or indirectly suppress IgE production and influence the development of allergy.

**Table 3:** **Allergen-specific IgE levels in the plasma of 49 infected and 46 uninfected patients with high and lower peripheral blood CD4^+^CD25^hi^ cell frequencies**.

	Median concentration (kUA/l) and interquartile range		
	House dust	Grass pollen	Birch pollen
***H. pylori* infected**	NS	*p* = 0.004	*p* = 0.002
High Treg frequencies	0.06 (0.03–0.14)	0.00 (0.00–0.00)	0.00 (0.00–0.00)
Lower Treg frequencies	0.05 (0.04–0.13)	0.02 (0.00–0.18)	0.01 (0.00–0.02)
**Uninfected**	NS	NS	NS
High Treg frequencies	0.03 (0.03–5.74)	0.00 (0.00–0.04)	0.00 (0.00–0.02)
Lower Treg frequencies	0.04 (0.02–0.09)	0.00 (0.00–0.03)	0.00 (0.00–0.02)

*Allergen-specific IgE data were stratified according to whether the patient had a high Treg response (>10% CD4^+^CD25^hi^ PBMCs) or if the frequency was below this level. A Mann–Whitney *U*-test analysis was performed to examine differences in the IgE responses of these groups. NS = no significant difference*.

### Mechanistic Experiments to Investigate the Role of Th1 and Treg Cytokines in the Suppression of IgE

**Figure 6:**
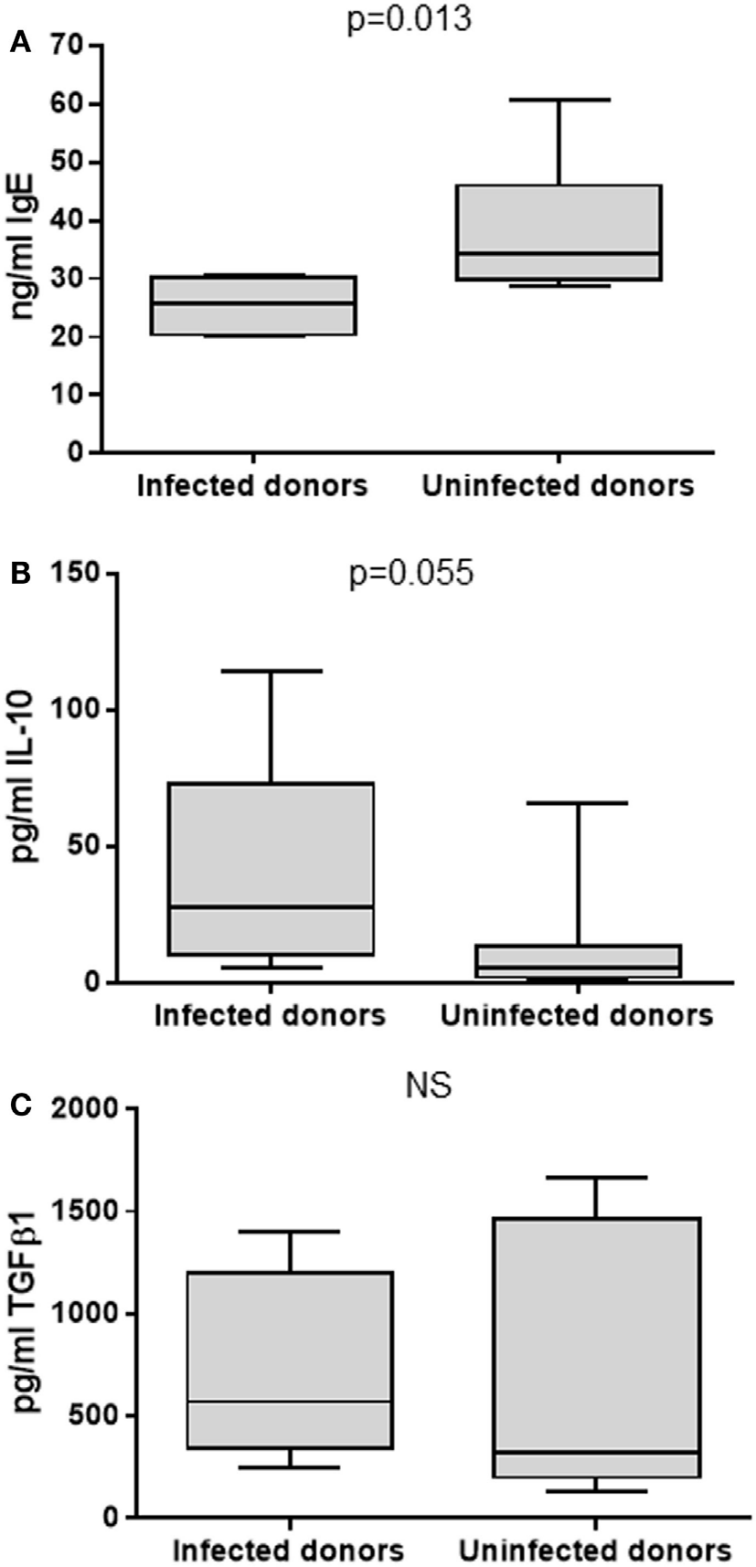
**Culture of PBMCs under Th2-skewing conditions**. PBMCs from 5 infected and 10 uninfected donors were cultured for 12 days before assaying the supernatants for total IgE **(A)**, IL-10 **(B)**, and TGFβ1 **(C)** by ELISA. NS = no significant difference.

To test the previously observed associations between *H. pylori* infection, serum IgE levels and abundance of peripheral blood Treg cells, an *in vitro* culture system was employed. PBMCs from 5 infected and 10 uninfected healthy donors (all without ulcers) were cultured under Th2-skewing conditions [with IL-4 and rCD40L (75)] for 12 days. Total IgE, IL-10, and TGFβ1 concentrations in the culture supernatants were then measured by ELISA. These culture conditions were used to investigate the hypothesis that PBMCs from *H. pylori*-positive donors would be inherently less able to respond to the Th2 stimulation compared to those from *H. pylori*-negative donors, possibly due to IL-10 or TGFβ inhibition of IgE production *in vitro*. The culture system provided an opportunity to investigate the consequence of adding or selectively blocking IL-10 and TGFβ. From the *in vivo* data, we hypothesized that blocking IL-10 would increase IgE production more markedly when the PBMCs were from *H. pylori*-positive donors. As anticipated, IgE concentrations were lower for the infected donors compared to those who were not infected (medians 25.8 and 34.2 pg/ml, respectively; *p* = 0.013; Figure 6A). The concentrations of IL-10 were fivefold higher in the supernatants of PBMCs from the infected donors (*p* = 0.055; Figure 6B), but there were no differences in the concentrations of TGFβ1 (Figure 6C).

**Figure 7:**
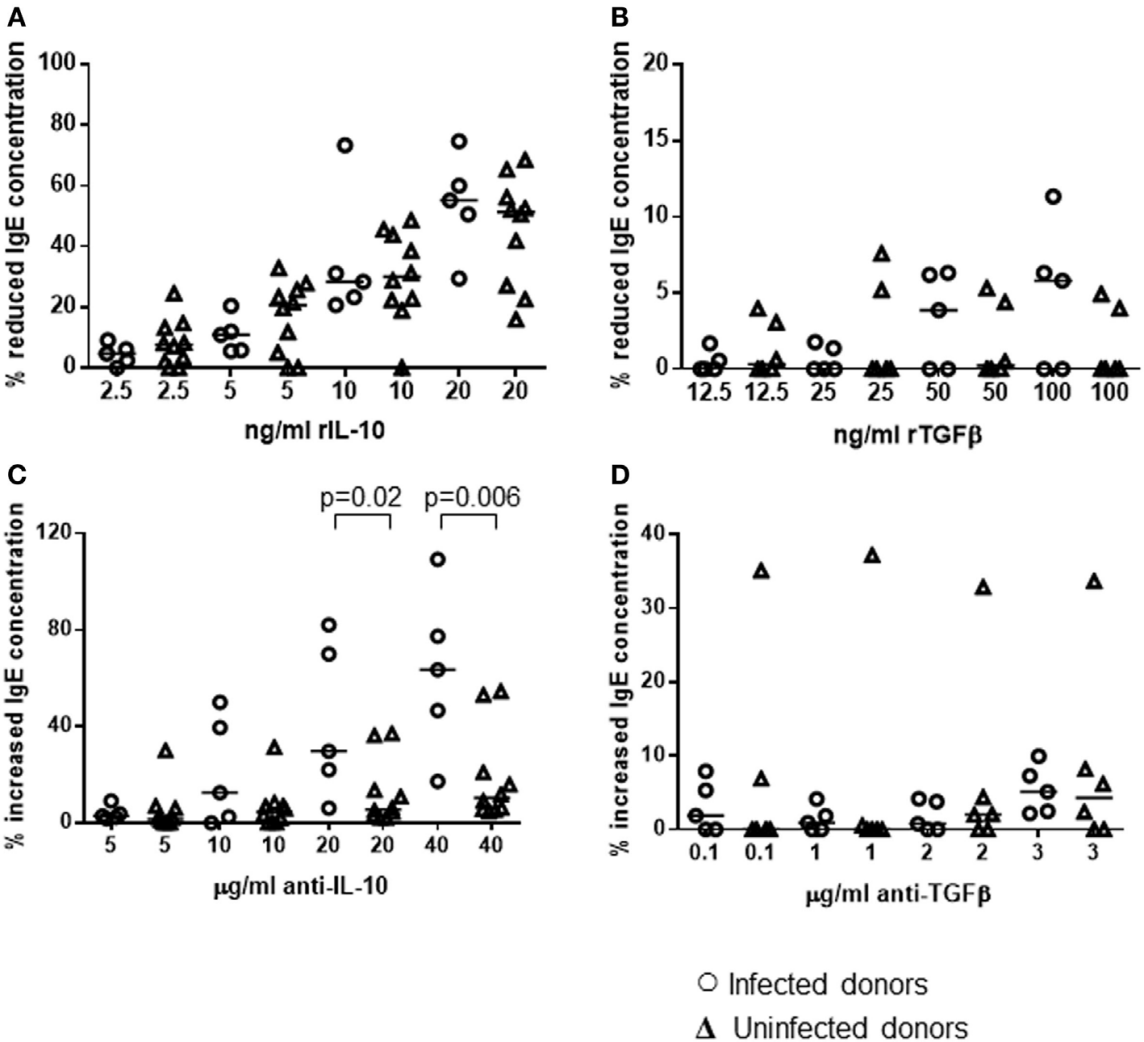
**Influence of recombinant IL-10 and TGFβ or their blocking antibodies on the *in vitro* IgE response of PBMCs from *H. pylori*-infected and -uninfected donors**. PBMCs from 5 infected and 10 uninfected donors were cultured for 12 days under Th2-skewing conditions, with the addition of rIL-10 **(A)**, rTGFβ **(B)**, anti-IL-10 **(C)**, or anti-TGFβ **(D)** blocking monoclonal antibodies. Equivalent volumes of medium were added to further wells as controls for recombinant cytokines; equivalent concentrations of isotype control antibodies were added as controls for the cytokine-blocking antibodies. Total IgE concentrations were assayed by ELISA and the percentage change was calculated for each individual with respect to the control cultures. Points represent the results from individuals and horizontal lines depict the median.

The cultures were also performed in the presence or absence of varying concentrations of recombinant IL-10, TGFβ1, or antibodies known to block the function of these cytokines. A range of concentrations was used so that we could investigate if there was a dose–response effect. Comparing each treatment to the non-treated control for each donor, the percentage decrease in total IgE resulting from the addition of recombinant cytokines was calculated. Adding the recombinant cytokines, at a range of concentrations reported previously to potently interfere with human T-helper cell activity (76, 81), caused a dose-dependent reduction in IgE secretion by PBMCs from both *H. pylori*-positive and -negative donors (Figures 7A,B). rIL-10 appeared to have a more potent effect than rTGFβ. Adding as little as 10 ng/ml rIL-10 resulted in a median 31% reduction in IgE concentration in the cultures compared to controls without rIL-10 (*p* = 0.020). Adding 20 ng/ml rIL-10 resulted in a median 52% reduction in IgE (*p* = 0.001). In contrast, a significantly reduced IgE concentration could only be achieved using the highest concentration of 100 ng/ml rTGFβ (5% reduction compared to no rTGFβ controls, *p* = 0.041). No differences in IgE response could be found between the PBMCs from *H. pylori*-positive and -negative donors in these assays however.

Adding IL-10 mAb, at a range of concentrations reported previously to block cytokine activity (77, 78), resulted in increased IgE production by PBMCs from both groups (*H. pylori*-positive and -negative donors), compared to cultures with the equivalent concentration of isotype control antibodies (Figure 7C). Adding anti-TGFβ had very little effect (Figure 7D). The difference in the size of the effect from IL-10 blockade between *H. pylori*-positive and -negative donors was statistically significant (20 μg/ml: 24%, *p* = 0.02; 40 μg/ml: 53%, *p* = 0.006). Therefore, we had confirmation that blocking IL-10, which was present at higher concentrations in the isotype control cultures of PBMCs from *H. pylori*-positive donors (Figure 6B), had a pronounced suppressive influence on IgE production *in vitro*.

## Discussion

This study aimed to further investigate the inverse association between *H. pylori* infection and allergy in humans, and fill an important gap in current literature by exploring the potential mechanisms behind this. We were able to demonstrate a link between IL-10-secreting peripheral blood Treg cell numbers and the concentration of IgE in the plasma of infected patients, and we also showed that IL-10 produced by leukocytes from those with *H. pylori* played an important role in controlling IgE production *in vitro*.

Our previous work found an increased Treg and Th1 response in the *H. pylori*-infected human gastric mucosa (48) and higher frequencies of Tregs in the peripheral blood of infected patients (68). These cell types have previously been implicated in the suppression of Th2 responses and allergy. We hypothesized that increased levels of Treg cells and/or Th1 cells in the circulation influence immune responses at extra-gastric sites in the body and may play a role in preventing an elevated IgE response. Initially, we compared the IFNγ, IL-10, and TGFβ1 responses of PBMCs to stimulation with *Hp* lysate antigen. Enhanced concentrations of IFNγ [as shown previously (82)] and IL-10 were found in the PBMC supernatants from infected patients, indicating that peripheral blood Th1 and Treg responses were increased. Cells from both *H. pylori*-positive and -negative patients secreted IFNγ and IL-10 in response to *Hp* lysate antigens, indicating that the response was not antigen specific. Flow cytometry was therefore used to determine whether increased frequencies of Th1 and Treg cells were present in the blood of infected patients.

Our experiments employed detection of CD69 expression, an early activation marker (83), as a tool to identify IFNγ^+^CD4^+^ Th1 cells that were responsive to *Hp* stimulation. This could be a consequence of antigen-specific reactivation of memory cells or T-cell receptor-independent immune activation. In agreement with the work of Lundgren et al. (84), peripheral blood CD4^+^ cells secreted IFNγ when stimulated with *Hp* antigen; however, there were no differences between the samples from infected and uninfected donors. The response is therefore unlikely to be due to reactivation of *H. pylori*-specific memory cells, but could arise because of shared or cross-reactive antigens with other bacteria, or because ligands for pattern recognition receptors were present in the antigen preparation (85).

In agreement with our previous work, and that of others, we found higher levels of CD4^+^CD25^hi^ lymphocytes among PBMCs from the infected donors (68, 84). It was necessary to determine if these populations could be classed as Tregs by using a selection of cellular markers, and a large proportion of the CD4^+^CD25^hi^ events expressed FOXP3 and/or IL-10. Since enhanced FOXP3^+^ Treg levels are present in the infected human gastric mucosa (48, 49, 51), we were surprised to find no difference in the frequencies of FOXP3^+^CD4^+^CD25^hi^ PBMCs, but this result was confirmed using RT-qPCR. FOXP3 is not a completely reliable marker for human Tregs, since its expression can be induced in activated cells that lack suppressive function, and not all Treg populations are FOXP3^+^ (56, 86). Interestingly, we were able to show increased levels of CD4^+^CD25^hi^IL-10^+^ lymphocytes in the PBMCs of infected patients. When stimulating PBMCs with *Hp* lysate antigen, a higher proportion of the Tregs from *H. pylori*-positive patients expressed IL-10. There was no effect on the CD4^+^CD25^hi^ population from uninfected patients, indicating the presence of a *H. pylori*-specific circulating Treg response. We were also able to confirm this by RT-qPCR, where higher levels of *IL10* mRNA were found in PBMCs from the infected patients. This agrees with several previous reports of *H. pylori*-specific IL-10-secreting CD4^+^ cells in the blood (87–89). We previously found increased levels of IL-10^+^FOXP3^+^ Tregs in the *H. pylori*-infected human gastric mucosa, cellular FOXP3 expression was of high level and could be confirmed by RT-qPCR (48). Our data suggest that slightly different Treg populations may be present in the blood, and these could include a higher proportion of FOXP3^−^ subtypes, such as Tr1 cells (which are IL-10^+^) (90) and FOXA1^+^ cells (associated with IFNβ responses) (91), since *H. pylori* stimulates the production of IFNβ by gastric epithelial cells (92). Recently, we were able to show that significantly higher proportions of peripheral blood Tregs from infected patients expressed the chemokine receptor CCR6 and that high concentrations of its ligand, CCL20, were present in infected human gastric mucosal tissue. The CCL20–CCR6 axis was shown to play an important role in the migration of Tregs to the infected gastric mucosa (68). Some studies have suggested that CCL20/CCR6 interactions are important in the development of Th2 responses and allergy (93, 94). It may therefore be the case that CCR6^+^ Tregs in the circulation of those infected with *H. pylori* are more capable of migration to the site of allergic responses.

Increased concentrations of IL-10 (but not IFNγ) are present in the serum of *H. pylori*-infected patients (95), and we have found high levels of *Hp*-specific IL-10-secreting Tregs in the circulation. This makes it a likely possibility that such cells could cause bystander suppression of other unrelated extra-gastric immune responses *via* IL-10 secretion. Since the major function of Tregs is to prevent autoimmune disease caused by an overzealous immune response (58), we hypothesized that the *H. pylori*-induced Treg response is involved in protection against allergy. Increased IL-10 responses have been postulated as a mechanism behind *H. pylori*-mediated protection from allergy in humans (96); however, Cam et al. found that PBMC IL-10 production was not influenced by *H. pylori* status or presence of atopy in children (97). Because of these controversies, we examined the relationship between plasma IgE concentrations and the frequency of IL-10^+^ CD4^+^CD25^hi^ Tregs among PBMCs in infected and uninfected individuals. No significant differences in IgE concentrations (total or antigen specific) were found between the groups of infected and uninfected donors. This may be due to collecting samples from patients with GI symptoms, where only a small proportion had concentrations of serum IgE above the normal range, and none had reported that they suffered from allergies or asthma. When associations between Treg frequency and plasma IgE concentration were examined, as anticipated from the literature (76, 98), higher Treg responses coincided with low IgE levels. This trend was exaggerated among the *H. pylori*-positive group, which contained more individuals with high Treg frequencies accompanied by lower concentrations of total, grass pollen-, and birch pollen-specific IgE. The trend was not statistically significant among the uninfected group, indicating that stronger Treg responses in *H. pylori* infection may suppress IgE levels. We acknowledge that as the samples were collected throughout the year, the data are likely to be confounded by seasonal variation in exposure to pollens. Regardless of this, we were able to detect significant differences in the relationship between IgE and circulating Tregs between the infected and uninfected groups.

Blaser et al. reported stronger protective associations between childhood asthma and more virulent CagA^+^ *H. pylori* infections (7, 9). Our data supported this finding, since those infected with *cagA*^+^ strains had threefold lower plasma IgE concentrations. A number of *H. pylori* virulence factors have been reported to influence the development of allergy in mouse models, including *H. pylori* neutrophil-activating protein (HP-NAP) (a potent stimulator of Th1 responses) and VacA (known to interfere with antigen presentation and to inhibit T-cell activation; also proposed to stimulate Tregs) (45, 99, 100). Strains which express the active i1 form of VacA are usually also *cagA*^+^ and those expressing the i2 form of VacA are usually *cagA*^−^ (101). This was indeed true in 39/49 of our clinical isolates and, since the *vacA* types also followed the same trend in the IgE data, we consider it likely that the reported stronger protection from CagA^+^ strains against childhood asthma could actually have been driven by VacA, with CagA acting as a marker for this. We previously found elevated IL-10 responses in the *cagA*^+^-infected gastric mucosa (48), but in the present study, we could not find a corresponding significant difference in the peripheral blood (data not shown). It has been suggested that *cagA*^+^ strains may elicit an increased gastric Treg response to modulate the heightened inflammation, but that there is also a pronounced effect on the migration of Tregs, which may have an impact on the populations found in peripheral blood (68, 102).

We performed experiments with an *in vitro* culture system to investigate the impact of IL-10 and TGFβ on IgE production in a well-controlled manner. The healthy donors who provided us with blood samples did not have different plasma IgE concentrations according to their *H. pylori* status (data not shown); therefore, these experiments allowed us to investigate the potential for their PBMCs to be influenced by a Th2-skewing treatment and produce IgE *in vitro*. The cells from the uninfected donors secreted significantly higher IgE concentrations, while producing markedly lower levels of IL-10. This implies that there were marked differences in the PBMC cell populations of infected and uninfected donors and is in agreement with the increased frequencies of IL-10-secreting Tregs among those with *H. pylori*. When rIL-10 was added to the cultures, in accordance with previous data, the PBMCs produced lower amounts of IgE (76). This occurred whether the cells were from infected or uninfected donors. When IL-10 blocking antibody was added, however, there was a significantly increased IgE response, which was especially marked when PBMCs were from infected donors. There were minimal effects from rTGFβ1 or TGFβ antibody blockade. These experiments demonstrate the important role of the *H. pylori*-induced IL-10 response in controlling IgE production.

We acknowledge that a weakness in this study comes from the fact that the blood samples did not come from allergic or atopic patients; however, we have gathered some interesting novel data that confirm and extend previous research on this topic. It is not possible to use an *in vivo* interventional approach in humans to study the role of IL-10 in the protective mechanism behind *H. pylori*-mediated protection from allergy. Our *in vitro* culture system provided some important clues, however, and enables us to move the project forward. Since we wish to understand the effects of *H. pylori* in protection against allergy, the best approach would be to perform an interventional study investigating the effects of antibiotic eradication therapy on allergic parameters and peripheral blood Treg frequencies. This would include looking for differences in IL-10-expressing populations in particular.

In summary, we demonstrated the presence of an increased frequency of regulatory T-cells in the peripheral blood of *H. pylori*-infected individuals. These cells tended to express IL-10, and high-level responses were associated with reduced plasma IgE concentrations. Using an *in vitro* culture system, we showed for the first time that PBMCs from *H. pylori*-infected donors had an enhanced ability to minimize IgE responses, acting in part *via* IL-10 since there was a 50% increase in IgE when IL-10 was blocked. Further *in vitro* work (not shown) found that CD4^+^CD25^hi^ cells, purified from PBMCs of infected and uninfected donors using magnetic bead separation, were able to suppress proliferation and IFNγ production by CD4^+^CD25^–^ effector T cells stimulated with anti-CD3/28 beads. This confirms that the cells have a regulatory function, and we are currently working to evaluate whether Tregs from infected individuals have any enhancement in suppressive activity. We also aim to carry out further experiments to elucidate the mechanisms, investigating the types of Tregs responsible (e.g., Tr1 and FOXA1^+^ cells), and are planning to investigate whether *H. pylori* exerts a long-lasting influence on the Treg and IL-10 response from early life, or if the immunological effects require continual life-long presence of the infection in the gastric mucosa. Understanding these concepts is going to be of paramount importance, considering the diminishing prevalence of *H. pylori* around the world, and newly reported hopes for an effective vaccine (103, 104).

## Author Contributions

KH, DL, AG, RK, JA, and KR designed the experiments; KH, DL, AG, RK, JW, WT, JR, ES, and KR carried out the experimental work; KH, DL, AG, RK, ES, KK, and KR analyzed the data; and KH, DL, KK, and KR wrote the manuscript.

## Conflict of Interest Statement

The authors declare that the research was conducted in the absence of any commercial or financial relationships that could be construed as a potential conflict of interest.

## Abbreviations

BFA, brefeldin A; conA, concanavalin A; CTLA-4, cytotoxic T-lymphocyte antigen 4; ECD, phycoerythrin-Texas Red; GITR, glucocorticoid-induced tumor necrosis factor receptor; IFN, interferon; IL, interleukin; mAb, monoclonal antibody; PC5, phycoerythrin cyanin 5.1; PE, phycoerythrin; PMA, phorbol myristate acetate; TGFβ, transforming growth factor-beta; Treg, regulatory T-cell.
